# Prevalence of dietary supplement use by gym members in Portugal and associated factors

**DOI:** 10.1186/s12970-020-00342-z

**Published:** 2020-02-24

**Authors:** João Ruano, Vitor Hugo Teixeira

**Affiliations:** 1 Faculty of Sciences, University of Porto, Porto, Portugal; 2 Faculty of Nutrition and Food Sciences, University of Porto, Porto, Portugal; 3 Research Centre in Physical Activity, Health and Leisure (CIAFEL), University of Porto, Porto, Portugal

**Keywords:** Nutritional, Supplementation, Exercise, Ergogenic

## Abstract

**Background:**

Although there seems to be an increasing interest in the use of dietary supplements in those who exercise recreationally and want to improve body composition, there is little published data regarding gym users and dietary supplement use.

**Methods:**

This cross-sectional study describes the prevalence and type of supplements used by gyms members, the reasons for using them and the information source using a self-administered online questionnaire.

**Results:**

Of the 459 participants (301 females) who answered the survey, 43.8% reported using dietary supplements. Users were more likely men (62.7% vs. 33.9%, *p* < 0.05), younger (32 ± 9 vs. 34 ± 11 years, *p* < 0.05) and trained more hours per week (6 ± 3 vs 4 ± 3 h, *p* < 0.05) than non-users. The most consumed supplements were proteins (80.1%), multivitamins and/or minerals (38.3%), sport bars (37.3%), branched-chain amino acids (BCAA’s) (36.8%) and n-3 fatty acids (35.5%). Men consumed more arginine, BCAA’s, creatine, glutamine, β-hydroxy-β-methylbutyrate (HMB), proteins, β-alanine, taurine, multivitamin/minerals, and carbohydrate supplements (*p* < 0.05). The most commonly cited reasons for the use of supplements were gaining muscle (55.7%), accelerating recovery (52.7%) and improving performance (47.3%). Men have more often referred increase strength, increase resistance, gain muscle mass, accelerate recovery and improve performance as reasons to use supplements than women (*p* < 0.05). Those who mentioned muscle gain as a reason were younger than those who did not (30.4 years vs. 33.7 years, *p* < 0.05). The sources of information most mentioned were registered dietitians (23.1%), internet (22.2%) and him/herself (16.6%). The majority (> 70%) of participants declared being well or very well informed about supplements, while only a minority (4%) felt very poorly or poorly informed. Most individuals purchased dietary supplements from the internet (56.2%) and supplement/health food stores (43.4%).

**Conclusion:**

This study concluded that gyms users are large consumers of dietary supplements, and are more likely to be men, young, use protein powders, aiming to increase muscle mass, obtain information from registered dietitians, consider themselves well informed and buy supplements online.

## Background

Compared to the impact that genetics fostered by an appropriate training program has on athletic performance, nutrition plays a relatively small role [1]. Athletic performance is influenced largely by genetics, training, and dietary intake. Elite athletes aiming to acquire a competitive edge also commonly use dietary supplements [2, 3]. Data suggests that athletes who report supplement use have more nutrient dense diets compared to those that do not use supplements [4].

The use of supplements is also increasing among non-athletes [5], who correspond to the bulk of the industry’s customers [2]. One of the places that emerged as the main place of consumption are the gyms [6, 7]. An increasing number of gym users is eager to take dietary supplements in order to increase lean body mass quickly [8], but without the advice provided by health professionals that athletes have available [7]. They often rely only on the information on the label, which may not be fully representative of the actual content of the supplement [8], or on the information provided by the manufacturer, that do not have to demonstrate supplements’ safety and efficacy [2]. The use of supplements should be done with caution and under supervision since adverse effects have already been reported [9]. It would be advisable that such supervision is performed by a health professional, such as medical doctors or registered dietitians, to clarify the consumer about the benefits and risks of using supplements, so that they can make an informed choice [10], and be responsible for the recommendation, to ensure a safety use [11].

Although there is much information on the use of dietary supplements by athletes [3, 12], there is fewer data available on their use by recreational users attending a gym. The use dietary supplements on recreational gym users appears to be influenced by their country [6, 13–16] and culture [1], therefore it might be interesting to provide country-specific data on dietary supplement use in recreational gym users and allow for targeted strategies to be drawn.

Our aim was to analyze the prevalence of consumption, to describe the type of supplements mostly used, the main reasons for its use and the source of advice of gym users in Portugal, contributing to the existing literature on the subject.

## Methods

### Participants

The sample size estimation (*n* = 372) was calculated using the following equation: *n* = z^2^ ∗ p ∗ (1-p)/W^2^, where n was the estimated sample size, z the normal distribution (defined as 1.96 for research with 95% confidence), p the estimated proportion of individuals who use supplements based on a study in the city of Porto (41%) [17], and W the study margin of error (5%). The Ethics Committee of the Faculty of Sport of the University of Porto was asked for approval, which was granted.

### Questionnaire

The self-administered questionnaire was based on and adapted from other previously used by our research group [4]. After being pre-tested for clarity and efficiency, the final version was converted to an online format using LimeSurvey®. The Association of Gyms and Academies of Portugal was contacted to disseminate the questionnaire through their members. In addition, some gyms directors were directly contacted and asked to send the questionnaire link by email to their members. According to the Portuguese Law, a gym is defined as a sports hall open to the public, equipped with strength training equipment, weight training or related activities, as well as those for the development, maintenance or recovery of physical condition, in particular for the practice of gymnastics, maintenance, aerobics or similar activities. Before completing the questionnaire, participants gave their informed consent.

The questionnaire was comprised of 3 parts: (1) demographic and anthropometric characteristics (age, sex, education, employment, residence, health status, medication, alcohol consumption and smoking habits), (2) exercise practice data (frequency, duration and type of exercise, in the gym and outside the gym), and (3) use of dietary supplements in the previous year (type of supplements, reasons of use/non-use, source of information, degree of satisfaction, place of purchase).

In accordance with previous studies [5, 10], supplements surveyed in this study correspond to the definition of Directive 2002/46/EC (“a product intended to supplement the normal diet, consisting of a concentrated source of a nutrient or of other substances that have a nutritional or physiological effect, in a simple or combined form, commercialized in dosed formulas, capsules, tablets, pills and other similar forms, bags of powder, vials of liquid, dropper bottles and other similar forms of liquids and powders, which is taken in small, quantified amounts”).

In order to help athletes, closed-ended options were available to identify the type of supplements taken (33 options), the main reasons of use (13 options) and the sources of information (13 options). The participants were able to choose more than one answer, if applicable. Furthermore, in all 3 topics, an additional open-ended question was included to allow answers other than those provided in the given list.

### Statistical analysis

After data collection, statistical analysis was performed with statistical software SPSS 24 for Windows (SPSS Inc., Chicago, IL, USA). Descriptive statistics was performed using frequencies and percentages for categorical variables, means for normally distributed continuous variables and medians for non-parametric continuous variables. To test for normality, the Kolmogorov-Smirnov test was used. The t-test was used when data was normally distributed and the Mann-Whitney test was used when not. Chi-square tests were used to identify associations between supplement use and the categorical variables, and Pearson’s correlation was performed for continuous variables. Statistical significance was set at *p* < 0.05.

## Results

### Sample characterization

By the end of the data collection (6 months) 644 questionnaires were filled, 71.3% of which were considered complete. Out of the 459 participants of the survey, 301 were female (65.6%) and 158 were male (34.4%), with a mean age of 33 ± 10 years (Table 1). The majority (71.5%) of the respondents had a higher education degree, were currently employed (77.7%) and were single (55.3%). The geographical distribution of the respondents revealed disproportionate participation of residents in the Porto district (31.2%). Most of the sample reported not consuming alcoholic beverages (65.4%) and did not smoke at the time of the survey (86.3%). In addition, the vast majority of respondents did not report any chronic disease (86.5%) and was not taking any type of medication (74.3%). Participants trained, on average, 4 times per week and spent 5 h training weekly. The main exercises performed were strength training (80.4% in men, and 57.5% in women), followed by functional training (24.7%) and CrossFit (20.9%) in men, and gymnastics (34.9%) and functional training (28.9%) and body jump (28.9%) in women. Furthermore, most of the participants (75.6%) practiced another type of exercise outside the gym.

**Table 1:** Sociodemographic and lifestyle characteristics of all participants and of supplements users

	Percentage of total population (*n* = 459)	Percentage of supplement users (*n* = 201)	P
Gender			
Male	34.4	49.3	< 0.05
Female	65.6	50.7	
Age Group			
18–25	25.6	26.3	< 0.05
25–35	37.8	42.9	
35–46	25.8	25.8	
46–56	6.5	2.5	
56–67	3.4	2.0	
67–77	0.9	0.5	
Education			
No schooling	0.2	0	0.418
4th year	0.2	0	
9th year	3.7	2.5	
12th year	24.4	23.4	
Bachelor’s Degree	1.5	1.5	
Graduation	46.4	45.3	
Master	21.4	23.9	
PhD	2.2	3.5	
Alcohol intake			
No	65.4	66.7	0.603
Yes	34.6	33.3	
Smoke status			
No	86.3	87.6	0.479
Yes	13.7	12.4	
Total time of exercise per week			
< 5 h	63.7	37.0	< 0.05
5–10 h	31.6	52.5	
10–15 h	3.3	7.5	
> 15 h	1.3	3.0	

### Supplement use

Less than half (*n* = 201, 43.8%) of the gym users reported having used at least one dietary supplement in the previous year. Men were more likely to use dietary supplements than women (62.7% vs. 33.9%, *p* < 0.05). It was also found that supplement consumers were younger when compared to non-consumers (32 ± 9 years vs. 34 ± 11 years respectively, *p* < 0.05) and trained more hours per week (6 ± 3 h vs 4 ± 3 h, *p* < 0.05). Other factors, such as education, alcohol intake, smoking status, and disease history, had no significant associations with supplement use.

**Fig. 1:**
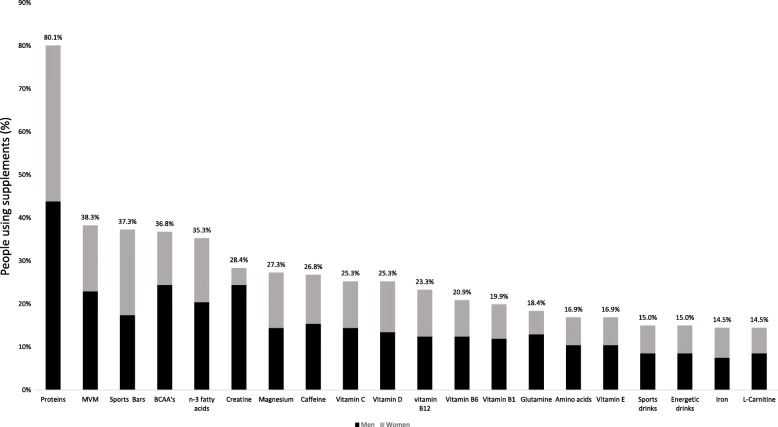
Most used supplements by nutritional supplement consumers; BCAA: branched-chain amino acids; MVM: Multivitamin and/or minerals supplements

The most widely consumed supplements (Fig. 1) were protein powders (80.1%), followed by multivitamins and/or minerals (38.3%), sport bars (37.3%), branched-chain amino acids (BCAA’s) (36.8%) and n-3 fatty acids (35.5%). A significantly higher number of men compared to women consumed arginine (12.1% vs 2%, *p* < 0.05), BCAA’s (49.5% vs 24.5%, *p* < 0.05), creatine (49.5% vs 7.8%, *p* < 0.05), glutamine (26.3% vs 10.8%, *p* < 0.05), HMB (9.1% vs 1%, *p* < 0.05), proteins (88.9% vs 71.6%, *p* < 0.05), β-alanine (8.1% vs 1%, *p* < 0.05), taurine (13.1% vs 2.9%, *p* < 0.05), multivitamin and/or minerals (46.5% vs 30.4%, *p* < 0.05) and carbohydrate supplements (16.2% vs 2.9%, *p* < 0.05). The supplements with the highest degree of satisfaction were vitamin C and proteins, with 40.4 and 36.9% of respondents, respectively, considering to be very satisfied from the results obtained when consuming these supplements. β-alanine, β-carotene, sport gels and ginseng had a percentage above 60% for non-satisfaction.

### Reasons to use

**Fig. 2:**
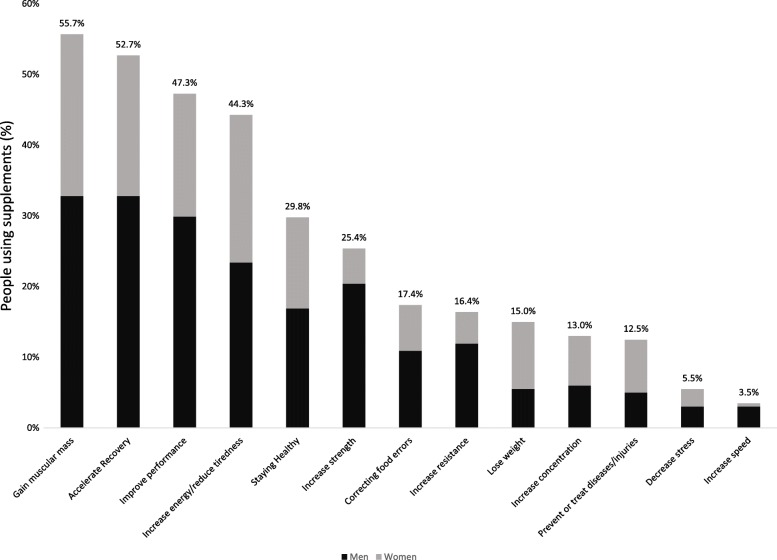
Reasons for supplement use referred by nutritional supplement consumers

The most cited reasons for consumers to use supplements (Fig. 2) were gaining muscle (55.7%), accelerating recovery (52.7%) and improving sports performance (47.3%). Increasing strength (41.4% vs. 9.8%, *p* < 0.05), increasing resistance (24.2% vs. 8.8%, *p* < 0.05), gaining muscle mass (66.7% vs. 45.1%, *p* < 0.05), accelerating recovery (66.7% vs. 39.2%, *p* < 0.05) and improving performance (60.6% vs. 34.3%, *p* < 0.05) were more often referred by males than by females. Those who reported that gaining muscle was a reason to use supplements were younger than those who did not (30.4 vs. 33.7 years, *p* < 0.05). Individuals who did not report ingesting dietary supplements declared that the reasons for not taking them were having already a balanced and adequate diet (67.4%) and ignoring the effect of the intake (29.1%). Men reported more often than women not being sure that dietary supplements would improve performance (10.2% vs. 3%, *p* < 0.05).

### Sources of information

**Fig. 3:**
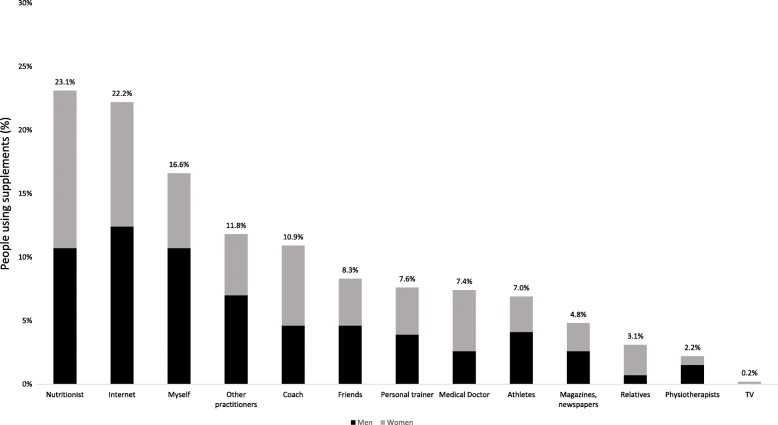
Sources of information referred by nutritional supplement consumers

The sources of information (Fig. 3) most referred by consumers of supplements were registered dietitians (23.1%), the internet (22.2%) and him/herself (16.6%). Men reported more often acquiring information from registered dietitians (31.0% vs 18.9%, *p* < 0.05), fitness coaches (11.4% vs 5.6%, *p* < 0.05), friends (13.3% vs 5.6%, *p* < 0.05), himself (31.0% vs 9.0%, *p* < 0.05), other sportsmen (20.3% vs 7.3%, *p* < 0.05), physiotherapists (4.4% vs 1.0%, *p* < 0.05) and on the internet (36.1% vs 15.0%, *p* < 0.05) than women did. Almost half of respondents (47.8%) considered themselves well informed, while 22.9% declared to be very well informed about the use and effect of supplements. Only a minority stated that were very poorly informed (0.5%) and poorly informed (3.5%).

### Purchase information

It has been found that dietary supplements were mainly purchased through the internet (56.2%), with supplement/health food stores being the second place of choice (43.4%). Men preferred to acquire dietary supplements through the internet (67.7% vs. 45.1%, *p* < 0.05), while women were more likely to buy them often in pharmacies (23.5% vs. 15.9%, *p* < 0.05) and supermarkets (2% vs. 11.8%, *p* < 0.05).

## Discussion

This study corroborates that individuals attending gyms are major consumers of dietary supplements. The prevalence of dietary supplements use we found (44%) is within what has been described in most studies in gym users (36–56%) [7, 8, 15], but much lower than what was observed in countries like the USA (84.7%) [18, 19] and Brazil (64.7%) [6]. The discrepancies in the reported prevalence rates may be related to sociodemographic and cultural characteristics, the type of gyms included or methodologic aspects, namely what was considered to be a supplement and the method of data acquisition [13]. There appears to be a trend towards an increase in the consumption of supplements in gyms users in Portugal, which was 25% in 2010 [20] and 41% 2 years after [20], but still lower than we have found for elite athletes (66%). This confirms the higher rate of supplement use in athletes than in regular gym members also found by others [14]. Although the role of gender as a determinant of supplement use is not clearly established, we found that supplement consumption was more prevalent among men, consistently with previous studies [4, 5, 7, 8, 13, 15].

Protein and amino acid where the most common type of supplement consumed, resembling other studies [7, 8, 19]. This finding can be explained by the importance that optimal protein intake has in increasing muscle mass [21] and the convenience of supplements [22]. It is not always feasible to ingest an adequate amount of protein exclusively from food, due to difficulties in preparation or transportation, lack of time or the volume needed to reach optimal doses [23]. Supplements also represent an easy way to increase protein intake in out-of-home snacks, promoting a more equitable distribution throughout the day, with advantages for muscle synthesis [24]. The difference in the intake of protein supplements between men and women was small, which is consistent with the fact that both sexes referred muscle hypertrophy as the main reason for the use of supplements. However, consistent with others findings [15, 18], men still reported more than they intended to increase muscle mass, which helps explain their increased use of other supplements supposedly involved in increasing muscle mass, such as creatine, HMB or BCAAs, despite lack of evidence for the latter two [1].

Protein powders, BCAAs supplements and multivitamins and/or minerals were the most consumed supplements, confirming previous findings [7, 13, 15], and also those with which consumers reported to be more satisfied with their use. The lack of scientific data to support most of the alleged ergogenic properties highlights the importance of the placebo effect on self-perceived efficacy of supplements and vulnerability to claims [25]. Although the supplementation with vitamins and minerals is frequent in athletes [26] and gym users [8, 18, 19] it is not justified in most cases, because their requirements are easily satisfied with adequate energy and micronutrient-dense diet, which is often the case in most supplement users [4, 8].

Participants mentioned gaining muscle mass, accelerating recovery and improving performance as main reasons for consuming supplements, which is similar to others findings [7]. Men were more interested in increasing muscle endurance and muscle mass and also improving performance and recovery, but the biggest discrepancy was that men reported much more aiming to increasing strength than women (41.4% vs. 9.8%, respectively). Earlier research [7] showed that men refer more performance-enhancing reasons (speed, strength, power, muscular hypertrophy), as opposed to women who tend to consume more health-related supplements (prevention of nutritional deficiencies or illness). However, despite omega-3 supplements (29.4%), multivitamins/minerals (30.4%) and sport bars (39.2%) being the top choices of women in our study, the use of protein powders was much higher (71.6%). Considering that the majority of women surveyed use protein powder, it could be reasoned that women perceive protein powder as a beneficial tool to improve body composition. This might account for the similar frequency of protein supplement use between men and women in this and other studies [6]. Reason for supplement consumption also varies according to age; users who aimed to gain muscle were younger, while older consumers referred health-related reasons [7, 8, 17]. Different reasons are listed by athletes and gym users to consume supplements. While the former refers that the main reason is to accelerate recovery [26], the latter do it mainly to increase muscle mass. This may explain why protein supplements were the most used dietary supplements by gym users but were not superior to multivitamins/minerals, sport drinks and magnesium supplements in Portuguese elite athletes [26].

The large majority of participants (> 70%) declared being well or very well informed about dietary supplements, while only a minority (4%) felt very poorly or poorly informed. Similarly to what was previously described [6, 13, 15], there was a high dependence on the internet as a source of information and place of purchase. However, our data points to a higher reliance in registered dietitians (22%) compared to the majority of studies so far, where only 10 to 19% of the participants referred to this health professional as a source of information [7, 8, 12, 15, 27]. This is consistent with what was previously found in Portugal, both for gym users [17] and elite athletes [4], in which more than half of respondents reported having health professionals as the main advisors. In other studies, supplements intake was self-prescribed [8, 13] or recommended by fitness instructors or co-athletes [5, 6].

Our study has inherent limitations, which should be acknowledged, the main one concerning the method of data collection (a self-administered online questionnaire) and the sample being of convenience. Two thirds (65.6%) of our sample were female, which differs from other studies [7, 8, 17, 20], and may reflect an increasing number of women exercising in gyms or greater interest in participating in the study. The majority (75%) of the respondents of this survey had a university degree, which may reflect the high level of education of those attending gyms, due to the recognition of associated health benefits [8, 17], or the greatest readiness to participate in studies.

## Conclusions

In conclusion, our study found that gyms users are large consumers of dietary supplements, mainly men. Protein powders, BCAAs supplements and multivitamins and/or minerals were the most consumed supplements and gaining muscle mass, accelerating recovery and improving performance were the main reasons for consuming them. We observed a high dependence on the internet as a source of information. This generalized consumption of supplements occurs despite the scarce evidence of its effects and the lack of knowledge of pre-existing nutritional deficiencies. Sports nutrition experts should provide scientifically correct information about the benefits and risks of using supplements, so that consumers can make informed choices, and encourage the role of a balanced diet in achieving their specific goals.

## Data Availability

Please contact author for data requests.

## Abbreviations

BCAA’s: Branched-chain amino acids
HBM: β-hydroxy-β-methylbutyrate
